# Anatomical Substrates of the Alerting, Orienting and Executive Control Components of Attention: Focus on the Posterior Parietal Lobe

**DOI:** 10.1371/journal.pone.0050590

**Published:** 2012-11-30

**Authors:** Xuntao Yin, Lu Zhao, Junhai Xu, Alan C. Evans, Lingzhong Fan, Haitao Ge, Yuchun Tang, Budhachandra Khundrakpam, Jian Wang, Shuwei Liu

**Affiliations:** 1 Research Center for Sectional and Imaging Anatomy, Shandong University School of Medicine, Jinan, Shandong, China; 2 McConnell Brain Imaging Centre, Montreal Neurological Institute, McGill University, Montreal, Quebec, Canada; 3 Department of Radiology, Southwest Hospital, the Third Military Medical University, Chongqing, China; 4 National Laboratory of Pattern Recognition, Institute of Automation, Chinese Academy of Sciences, Beijing, China; Radboud University Nijmegen, The Netherlands

## Abstract

Both neuropsychological and functional neuroimaging studies have identified that the posterior parietal lobe (PPL) is critical for the attention function. However, the unique role of distinct parietal cortical subregions and their underlying white matter (WM) remains in question. In this study, we collected both magnetic resonance imaging and diffusion tensor imaging (DTI) data in normal participants, and evaluated their attention performance using attention network test (ANT), which could isolate three different attention components: alerting, orienting and executive control. Cortical thickness, surface area and DTI parameters were extracted from predefined PPL subregions and correlated with behavioural performance. Tract-based spatial statistics (TBSS) was used for the voxel-wise statistical analysis. Results indicated structure-behaviour relationships on multiple levels. First, a link between the cortical thickness and WM integrity of the right inferior parietal regions and orienting performance was observed. Specifically, probabilistic tractography demonstrated that the integrity of WM connectivity between the bilateral inferior parietal lobules mediated the orienting performance. Second, the scores of executive control were significantly associated with the WM diffusion metrics of the right supramarginal gyrus. Finally, TBSS analysis revealed that alerting performance was significant correlated with the fractional anisotropy of local WM connecting the right thalamus and supplementary motor area. We conclude that distinct areas and features within PPL are associated with different components of attention. These findings could yield a more complete understanding of the nature of the PPL contribution to visuospatial attention.

## Introduction

Attention refers to both the preparedness for and selection of certain environmental or mental aspects [1]. Because of the limited capacity of the brain to handle information, the appropriate selection of information for processing becomes especially critical in our daily life. Although many competing theories have proposed a number of potential components of attention, growing consensus indicates that there are three key subsystems of attention, i.e. alerting, orienting, and executive control [2]–[7]. Briefly, alerting is defined as achieving and maintaining a state of high sensitivity; orienting refers to the selection of sensory information; and executive control is involved with the processing of cognitively incongruent stimuli or conflict [8]. These components of attention network have been shown to differ in their functional anatomy [3], neural circuits [9] and neurochemical pathways [10].

In order to isolate the functional components of attention and to investigate their association, Fan and colleagues [11] invented the attention network test (ANT). Combining the cued reaction time (RT) and the flanker [12] task, ANT provides a means for exploring the behavioural reaction of the three attention components in a single integrated task. Using ANT as the behavioural task and vertex-based analysis, Westlye et al [6] reported that executive control was associated with cortical thickness in the prefrontal regions and temporoparietal junction, and alerting was negatively correlated with the cortical thickness of the left superior parietal region. By means of diffusion tensor imaging (DTI) and regions of interest (ROI) analysis, structure–function correlations were also found between alerting and the left posterior limb of the internal capsule, orienting and the splenium of corpus callosum, as well as executive control and the anterior corona radiata [5]. Although the subsistent relationships between attention components and the individual variances in brain cortical and subcortical structures were explored by these studies, it has rarely been investigated in an integrated manner, and very little is known about the specific organization and interaction between the grey matter and white matter (WM) engaged in attention function.

The posterior parietal lobe (PPL) has long been recognized as a neural substrate of visuospatial attention. With its extensive connectivity to cortical and subcortical regions in occipital [13], temporal [14] and frontal [15] lobes, PPL encompasses polymodal sensory convergence areas and plays key roles in attention function [16]–[18]. The dysfunction of PPL has been implicated in the pathophysiology of attention impairment, such as deficit/hyperactivity disorder [19], [20] and spatial neglect [21]–[24].

However, the roles of distinct parietal subregions on attention function are still highly debated, mainly due to the inconsistency among the attention models and the variations in the utilized imaging modalities. For example, alerting sometimes is termed as sustained attention or vigilance. Although there are slight differences between them, these functions are considered as part of a system controlling the intensity of attention, rather than its selectivity [23]. Based on lesion and imaging data, Posner and Petersen [2] pointed out that the right PPL might play a special role in maintaining an alert state. Some positron emission tomography (PET) studies also localized areas associated with sustained attention in the right superior parietal lobule (SPL) [25], [26]. However, Yanaka et al. [27] failed to detect PPL activation related to the warning stimuli, irrespective of the warning modality (visual or auditory), but found neural activation in the anterior cingulate cortex, thalami and pre-supplementary motor area.

On the other hand, orienting represents the characteristic of selective attention. Data from functional imaging studies manifested that bottom-up orienting, mediated by stimulus salience and/or relevance, was subserved by inferior parietal regions [28], [29], such as the angular gyrus (AG) [30]. Many electrophysiological studies have also shown that neurons in lateral intraparietal area respond to salient spatial stimuli with elevated activity [15], [31]–[33]. Importantly, one recent study found that the right IPL within lateral intraparietal area exerted the inhibitory effect over the contralateral homologous area, which might represent the neurophysiological mechanism of the well known asymmetry of visuospatial function [34]. In light of these pioneer studies, we speculated that stimulus-driven orienting might be underpinned by inferior parietal cortex and WM connecting bilateral parietal areas.

Finally, executive control is commonly measured using tasks in which there is an incompatibility between dimensions of the stimulus or response. Several recent studies have suggested that the temporoparietal junction in the right hemisphere responds to intersensory and sensorimotor conflict [35]–[37]. One case report found that patient with bilateral parietal lesions was impaired at filtering out competing distractors and discriminating behaviourally relevant objects [38]. However, despite extensive neuropsychological research [37], [39], the structural brain bases of executive control remain elusive.

Generally, it is proposed that human PPL is involved in three distinct cognitive functions: spatial perception, vision-for-action and visuospatial attention [23]. The taxonomy of alerting, orienting and executive control attention encompasses those parietal functions and integrates different attention components into one complete system. Controversy over the anatomical substrates of attention has highlighted the heterogeneity of this core cognitive operation and emphasized the complicated roles of distinct parietal subregions. Based on previous literature, we predict that distinct areas within PPL are engaged in different components of attention; and that the respective or interactive features of the parietal cortices and WM are associated with the individual variations in attention performance. To test these hypotheses, we measured brain cortical thickness, surface area and an array of DTI parameters in PPL subregions, and investigate their relationships with alerting, orienting and executive control performance of ANT in healthy young individuals.

## Materials and Methods

### Subjects

Healthy Chinese participants with ages ranging from 17 to 20 years were recruited from the local community. There were four inclusion criteria: (1) The participants were right-handed investigated by Edinburgh inventory [40], and had normal or corrected-to-normal vision acuity. (2) They had normal neurological exams and no history of neurological or psychological illness, or serious head injury. (3) There were no abnormal findings during brain magnetic resonance imaging (MRI). (4) The accuracy of ANT performance was not less than 80% and all the scores of ANT were positive. Forty volunteers took part in this study and 36 (22 males) participants satisfied these criteria. The qualified males and females did not differ in mean age (18.4±0.9 vs. 17.9±0.9 years) and education years (8.6±1.2 vs. 8.5±1.0 years). The study was approved by the Human Research Ethics Committees of the Shandong University School of Medicine. Written informed consent was obtained from all participants.

### Behavioural Task

A version of the ANT devised by Fan and colleagues [3] was adapted as the cognitive task for this study. Subjects were instructed to press a button as quickly and accurately as possible to make a left-right determination of the target, which was a leftward or rightward arrow at the center and flanked on either side by two arrows in the same direction (congruent condition), or in the opposite direction (incongruent condition). A cue (an asterisk) was presented before the appearance of target. There were three cue conditions: no cue (baseline), center cue (at the fixation for alerting), and spatial cue (at the target location for alerting plus orienting). The 6 trial types (3 cue conditions by 2 target conditions) were presented in a predetermined counterbalanced order in one block, which consisted of 36 trials plus 2 buffer trials at the beginning and lasted 5 min 42 s. Each subject performed a total of 6 blocks. All the subjects were trained before the formal operation. Stimulus presentation and behavioural response collection was performed using E-Prime (Psychology Software Tools, Pittsburgh, PA).

### Imaging Data Acquisition

DTI was carried out using a 3-T GE Signa scanner (General Electric Medical Systems, Milwaukee, WI, USA). The images were collected using diffusion-weighted imaging with an array spatial sensitivity encoding technique (ASSET) based on a single-shot echo-planar imaging sequence (TR/TE = 14000/75.1 ms, 96 × 96 matrix, FOV = 250 mm, 2.6 mm thick slices, no gap). The DTI scheme included the collection of 30 directions with non-collinear diffusion gradients (b = 1000 s/mm^2^) and 3 non-diffusion-weighted images (b = 0 s/mm^2^). From each participant 56 axial slices were acquired and the diffusion sequence was repeated 2 times to increase signal-to-noise ratio.

At the end of the DTI scans, a three-dimensional volume spoiled gradient-echo (SPGR) pulse sequence with 174 slices (TR = 6.5 ms, TE = 2.0 ms, slice thickness = 1.0 mm, matrix of 256×256, FOV = 256 mm, flip angle = 15°) was used to acquire the anatomical images for structural analyses.

### Behavioural Data Analysis

Firstly the total accuracy of each subject was calculated and the trials with incorrect responses or with RTs longer than 1500 ms or shorter than 200 ms were excluded to avoid the influence of the outliers. We also removed responses following erroneous ones to avoid post-error slowing effect. Since RTs were not normally distributed, we used median RT per condition as raw scores. Finally, instead of the conventional subtraction measure [3], we used ratio scores to definite the efficiency of three attention networks. The ratio scores, which had been used to explore the structure-behaviour correlations [6], [41], heritability [42] and attention impairment in diseases [43]–[45], would be more appropriate than RT scores in ANT studies, because the former could isolate the attention system from the overall RT. The formulas were as follows:
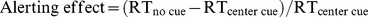


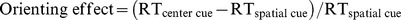


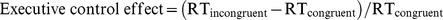



### MRI Data Analysis

The MR images were first processed with the CIVET MRI analysis pipeline (version 1.1.9) developed at Montreal Neurological Institute (MNI) to automatically extract and co-register the cortical surfaces for each subject. The main pipeline processing steps included: (1) The native three-dimentional structural MR image of each subject was corrected for non-uniformity using the N3 algorithm [46], and linearly registered into MNI152 standard space [47]. (2) Each brain volume was classified into gray matter, WM, cerebrospinal fluid and background using the INSECT algorithm [48]. (3) The Constrained Laplacian-based Anatomic Segmentation with Proximity (CLASP) algorithm was applied to generate a model of the cortical surface composed of 40962 vertices for each hemisphere [49]. (4) To obtain accurate cross-subject correspondences, the extracted hemispheric cortical surfaces were nonlinearly aligned to a hemisphere-unbiased iterative surface template [50]. (5) The aligned cortical surfaces were rescaled back to native space dimension using the inverse of the scaling parameters of the corresponding linear volumetric transformation matrix. (6) The cortical thickness was measured using the t-link metric of computing the Euclidean distance between linked vertices respectively on the inner and outer cortical surfaces [51].

**Figure 1:**
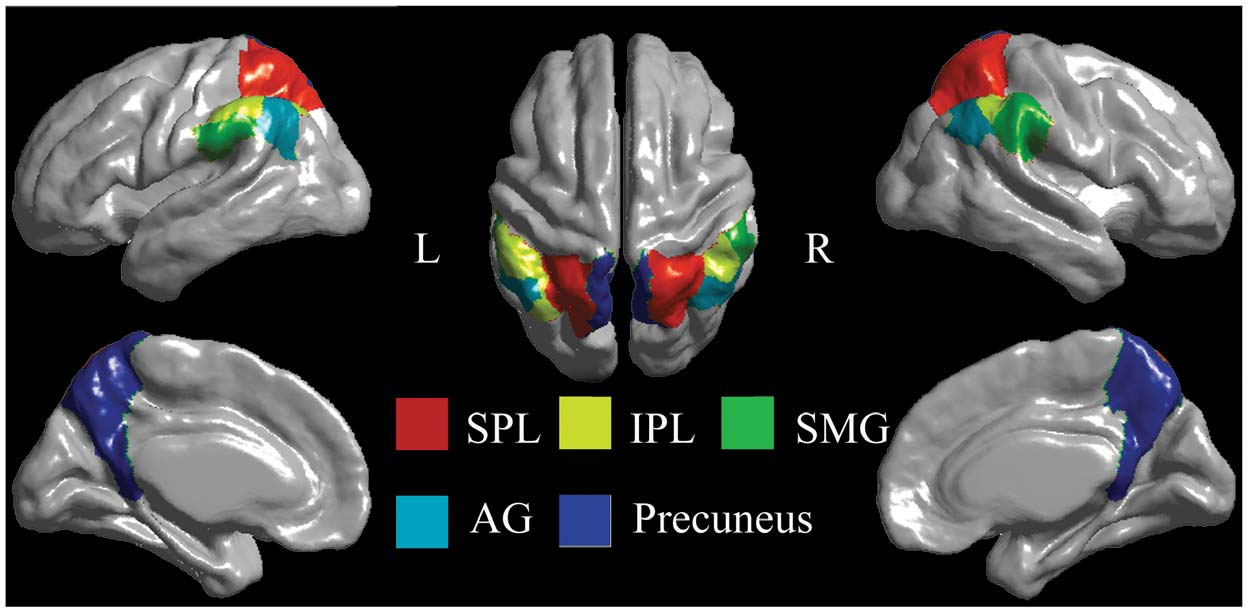
AAL parcellation of the PPL. The PPL surface is parcellated into 5 different gyral-based areas in each hemisphere. These areas are shown in different colors on the surface of the average brain across the sample. SPL, superior parietal lobule; IPL, inferior parietal lobule; SMG, supramarginal gurus; AG, angular gyrus; L, left; R, right.

In this study, we employed the automated anatomical labelling (AAL) template [52] to segment the PPL in each hemisphere into five partitions: SPL, IPL, supramarginal gyrus (SMG), AG, and precuneus (Figure 1). Note that the AAL template was originally defined on the MNI single brain and subsequently registered to the ICBM surface model. The mean cortical thickness was generated by averaging over all vertices within the same PPL subregion, while the surface area was computed by summing all the areas of the triangles included in that subregion on the mid-surface.

### DTI Data Analysis

The DTI data was pre-processed using FSL (University of Oxford, UK). Firstly the diffusion data were corrected for eddy currents and head motion and the two acquisitions were averaged. The averaged images were masked to remove skull and non-brain tissue using the FSL Brain Extraction Tool (BET) [53]. Then the diffusion parametric images were calculated using the diffusion tensor analysis toolkit (FDT) [54].

We then used tract-based spatial statistics (TBSS) [55] to test for local correlations between attention performance and fractional anisotropy (FA) in the WM. First, FA images for all subjects were non-linearly aligned to a study-specific, minimal-deformation target (MDT) brain and resampled to an isotropic 1 mm resolution. The group’s MDT brain was identified by warping all FA images in the group to each other [56]. Then, the mean FA image across all participants was calculated and used to generate a binary WM “skeleton” mask. The skeleton is formed by lines of maximum FA, assumed to correspond to the center of WM tracts. The FA threshold of 0.25 was chosen to restrict the skeleton to WM tracts. Each subject’s aligned FA data were then projected onto this skeleton. Note that no spatial smoothing was applied to the FA maps or skeleton values. Using the registration parameters and the distance map obtained for the FA images, the mean diffusivity (MD), radial diffusivity (RD, diffusivity perpendicular to the axon), and axial diffusivity (AD, diffusivity along the axon) images were also normalized into standard space and skeleton maps for these parameters were created.

Finally, we also used AAL template to segment the WM skeleton. To do this, T_1_-weighted structural image of MDT was first co-registered to the resampled b0 image in MNI space using a linear transformation. The transformed structural image was then mapped to the MNI single-subject MRI brain template using a nonlinear transformation. The resulting inverse transformation was then used to warp the AAL mask from the brain template to the “skeleton” brain in which the discrete labeling values were preserved by using a nearest neighbor interpolation method [57].

### Probabilistic Diffusion Tractography (PDT)

Voxels identified in the TBSS analysis were then used as seed masks for multi-fiber probabilistic tractography [58] in each subject’s native space. For each participant, PDT was run from each voxel in the seed mask to the whole brain and thresholded to include only voxels containing at least 50 samples (out of 5,000). The warpfields of nonlinear registration and the inverse versions were used for the translation between the original space and the standard space. These normalized individual tracts were then binarized and summed to produce group probability map.

### Statistical Analyses

The frequency histograms and Shapiro-Wilk tests showed that the behavioural data presented approximately normal distribution. To explore the relationships between the different ANT components, we correlated each of the ratio scores after partialling out gender and age. We then tested for main effects of gender on ANT scores and AAL-based variables using independent samples t-test. Linear relationships with ANT scores were investigated for all measures of the ten PPL labels (with gender, age and brain volume regressed out) in order to simplify comparisons across measures and regions.

In order to explore the unique contributions of the different measures, stepwise multiple regressions with each of the ANT scores as the dependent variable, age, gender and brain volume as confounding covariates, and regional cortical thickness, surface area, and FA as predictors were performed. The same procedure was repeated for MD instead of FA. Separate analyses were performed using FA and MD to avoid underestimates of contributions of these parameters because FA and MD are indexes reflecting the same diffusion eigenvalues [59]. Finally, Pearson’s correlations were performed between grey matter thickness/surface area and DTI parameters of WM in PPL subregions. The level of significance for all correlation analyses was set at <0.005, uncorrected, which was equal to p<0.05 with Bonferroni’s correction for multiple comparisons.

## Results

### Behavioural Data

The accuracy of ANT performance was 95.3%±3.4%, indicating that the participants understood the instructions and were able to respond reliably. There were no correlations with ANT scores on overall accuracy before and after partialling out gender and age. Therefore, the accuracy was not included as covariate in the subsequent analyses. The ratio scores of alerting, orienting and executive control effects as well as their correlations were summarized in Table 1. There were no gender differences in ratio scores and no significant correlations between ANT components. We did not find age correlations with any of the ratio scores across subjects.

**Table 1:** The ratio scores (Mean±SD) of attention components and their correlation coefficients (bold).

	Sample size	Alerting	Orienting	Executive control
Male	22	6.14±3.20	11.12±6.13	14.94±4.30
Female	14	5.77±2.85	10.36±4.09	14.42±3.71
t (*p*)		0.35 (0.73)	0.41 (0.69)	0.37 (0.72)
Total	36	5.99±3.04	10.82±5.37	14.74±4.03
Orienting	36	**−0.19** (0.29)		
Executive control	36	**0.15** (0.40)	**−0.01** (0.97)	

The effects of alerting, orienting and executive control are expressed in percent relative to the relevant baseline condition. The correlation analyses are adjusted for age and gender. t, the t value of independent samples t-test. The numbers in parentheses represent p values of statistical analyses.

### Cortical Thickness and Surface Area

Generally, males showed larger global mean cortical thickness (3.33±0.14 vs. 3.22±0.13 mm, p = 0.025), surface area (2416.76±90.12 vs. 2277.49±73.73 cm^2^, p<0.001) and brain volume (1.44±0.09 vs. 1.28±0.10 L, p<0.001) as compared to the females. Subsequent AAL-based analyses indicated that the cortical thickness of the left SMG, and the surface area of the right IPL, bilateral SPL and precuneus in men was significantly larger (p<0.005) than in women. Interhemispheric differences were not investigated here because it might be confounded by the asymmetry of AAL partitions between hemispheres.

Linear partial correlation analyses between ANT scores and cortical thickness or surface area in the 10 PPL labels when controlled for age, gender and brain volume indicated that orienting was negatively correlated with cortical thickness in the right AG (r = −0.49, p = 0.004). We also found a correlation between orienting score and cortical thickness of both the left (r = −0.44, p = 0.01) and right (−0.45, p = 0.01) SPL, but both failed to reach the statistical significance of 0.005. No significant findings emerged between cortical parameters and alerting as well as executive control performance (p>0.05).

### DTI

AAL-based correlation analyses revealed a significant negative correlation between orienting score and MD of WM skeleton underlying the right IPL (r = −0.49, p = 0.004), after controlled for age and gender. A positive relationship was also found between executive control performance and skeleton MD (r = 0.47, p = 0.005) as well as RD (r = 0.49, p = 0.003) in the right SMG. Nevertheless, no significant correlations were found between ANT scores and skeleton FA underlying the PPL (p>0.005).

**Figure 2:**
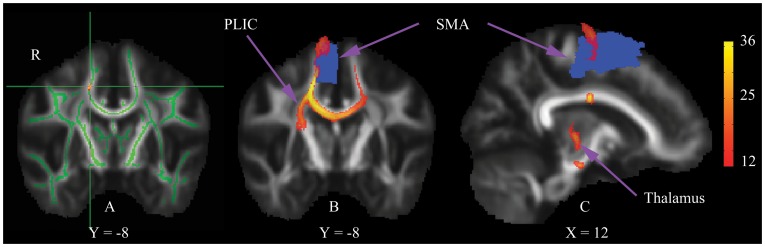
Local correlation between skeleton FA and alerting function (A), and probabilistic tractography from the alerting-related region (B and C). The cross center illustrates the peak MNI coordinate (x = 17, y = **−**8, z = 45) of the correlated region (Red-Yellow), which is overlaid on the standardized mean FA image. The mean FA skeleton is shown in green. The mask of the right supplementary motor area (SMA) comes from the transformed AAL template. The colorbar indicates the group tractography map with at least 1/3 of probability. PLIC, posterior limb of internal capsule; R, right.

Voxel-wise analysis indicated that alerting scores were associated with regional skeleton FA underlying the right frontal lobe (14 voxels; p<0.05, false discovery rate corrected) (Figure 2A). No significant cluster with ≥5 voxels was found for orienting and executive control performance. In order to assess the fiber pathways arising from the alerting-related region, we used the related cluster as seed mask for PDT. It generated longitudinal pathways, which passed through the posterior limb of internal capsule and reached towards the right supplementary motor area (Figure 2B) and thalamus (Figure 2C).

### Unique Effects of Cortical Thickness, Surface Area, and Diffusion Parameters

The results from the stepwise multiple regressions on orienting performance verified the above findings. Specially, for FA as one of the variables, the optimal model (F = 7.65, p<0.001, R^2^ = 0.61) included cortical thickness of the right IPL (β = 0.96, p = 0.001) and AG (β = −1.27, p<0.001), as well as local FA underlying the right IPL (β = 0.28, p = 0.02); for MD, the optimal model (F = 10.39, p<0.001, R^2^ = 0.68) also included cortical thickness of the right IPL (β = 0.92, p = 0.001) and AG (β = −1.22, p<0.001), as well as local MD underlying the right IPL (β = −0.41, p = 0.001). The similar multiple regression analyses for alerting and executive control were not conducted because of the lack of significant relationships to the cortical thickness and surface area during the aforementioned correlation analyses.

### The Relationship between Orienting and IPL Interhemispheric Connection

**Figure 3:**
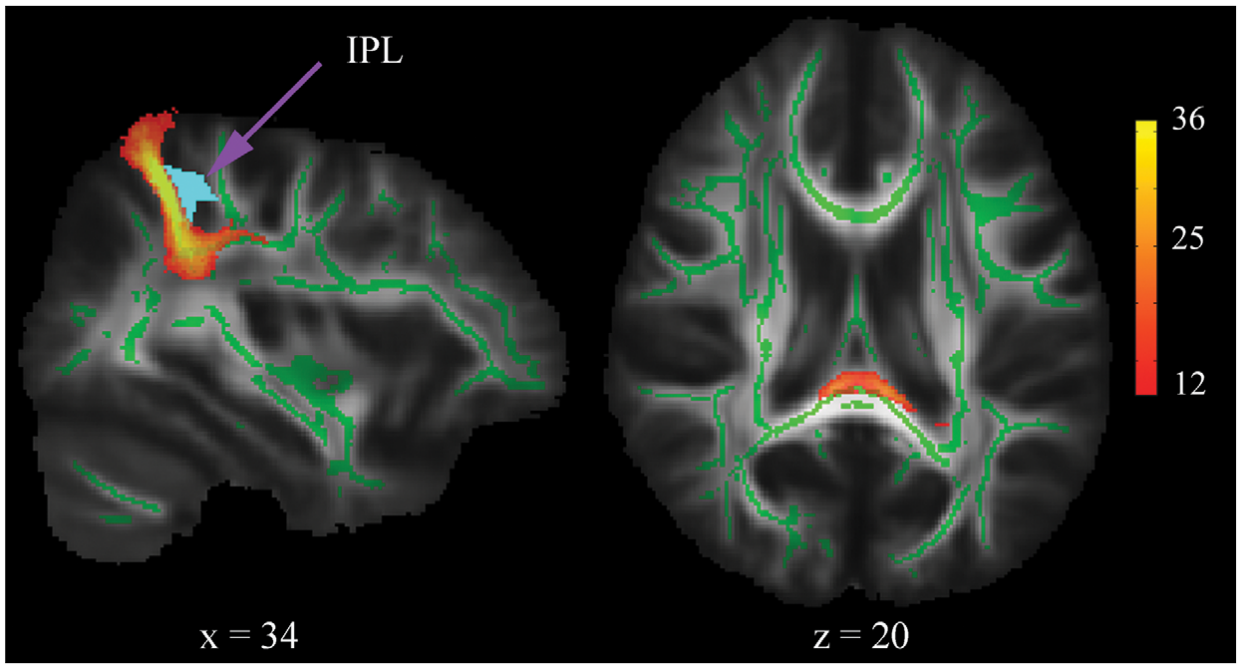
Sagittal and axial views of the group probability tractography map for WM connections of the bilateral inferior parietal lobules (IPLs). The lightblue mask represents the right IPL.

To confirm that the orienting function was associated with the WM pathways connecting the left and right IPL, we performed additional tractography analysis, in which the right IPL skeleton was chosen as the seed mask and the entire left IPL was designated as the termination mask. In this case, pathways originating from the seed mask would be terminated as soon as they enter the termination mask. As the seed mask was much broader than the alerting-related cluster, we thresholded the tracking map in each subject using 1250 (25%), instead of 50 samples. Then the individual tracts were binarized and summed to produce group probability map (Figure 3). The resulting map was arbitrarily thresholded to include at least one third of probability and multiplied with the DTI skeleton maps in order to extract the mean FA, MD, RD and AD values of individual tracts. Finally, we correlated these measures with the behavioural scores. The results were shown in Table 2. Intriguingly, the mean FA, MD and RD of WM connecting the bilateral IPLs were significantly correlated with orienting rather than alerting or executive control performance.

**Table 2:** Correlation coefficients (r) between attention performance and DTI parameters of IPL interhemispheric connecting fibers.

	FA		MD		RD		AD	
	r	p	r	p	r	p	r	p
Alerting	0.05	0.79	**−**0.09	0.61	**−**0.08	0.66	**−**0.06	0.75
Orienting	0.37	0.03	**−**0.38	0.02	**−**0.47	0.005	**−**0.02	0.92
executive control	0.12	0.95	0.18	0.31	0.13	0.45	0.14	0.42

The correlation analyses are adjusted for age and gender. p, the p values of the statistical analyses.

### Associations between Cortical Thickness, Surface Area, and Diffusion Parameters

Partial correlations between cortical thickness and other measures when controlled for age, gender and brain volume were shown in Table 3. Cortical thickness correlated negatively with surface area and FA in some of the 10 regions (p<0.005). These regional cortical thickness/FA relationships were primarily attributable to variations in RD. Conversely, correlations between surface area and the diffusion measures in each region were less significant and mainly appeared in the left SMG.

**Table 3:** The relationships of cortical thickness to the surface area (SA) and DTI parameters in PPL.

Regions	Hemispheres	SA	FA	MD	RD	AD
SPG	L	−0.33	−0.48**	0.27	0.42*	−0.12
	R	−0.16	−0.57**	0.34*	0.53**	−0.10
IPL	L	−0.49**	−0.39*	0.43*	0.47*	0.02
	R	−0.16	0.02	−0.01	−0.02	0.02
SMG	L	−0.49**	−0.50*	0.40*	0.54**	−0.10
	R	−0.63**	−0.21	−0.17	0.03	−0.11
AG	L	−0.29	0.15	0.24	0.07	0.24
	R	0.01	−0.33	0.25	0.42*	−0.12
Precuneus	L	−0.46*	−0.29	0.35*	0.39*	0.11
	R	−0.28	−0.60**	0.48**	0.62**	−0.09

The correlation analyses are adjusted for age, gender and brain volume. Single star (*) and double stars (**) represent the correlation was significant at p<0.05 and <0.005 (uncorrected), respectively. SPL, superior parietal lobule; IPL, inferior parietal lobule; SMG, supramarginal gurus; AG, angular gyrus; L, left; R, right.

## Discussion

The present study demonstrated that interindividual variations in the ability of spatial attention reflect variability in cortical thickness and WM integrity in distinct posterior parietal subregions. Specifically, the orienting performance was linked to the cortical thickness of the right IPL and AG, and the interhemispheric WM connections between the bilateral IPLs. Additionally, the executive control scores were significantly associated with the MD/RD of the WM underlying the right SMG. We found no significant correlations for parietal structures with the alerting effect. Instead, current study indicated that alerting may be partly mediated by the structural connectivity between the right supplementary motor area and the ipsilateral thalamus.

Our main finding was that variations of the orienting performance were associated with the cortical thickness and FA values of the right IPL in the multiple regression analysis. One caution is that IPL in AAL template occupies the anterio-inferior part of the lateral intraparietal sulcus adjacent to but not composed of the SMG and AG. Although the intraparietal sulcus has been designated as a crucial node of dorsal attention network in Corbetta’s dichotomy model [29], [60], literature built on the accumulated functional imaging experiments suggests that intraparietal sulcus also engages in automatically stimulus-driven spatial attention in both human [31], [61]–[63] and monkeys [15]. In fact, the lateral intraparietal area, whose activity could be biased by both bottom-up stimulus-driven factors and top-down cognitive influences, has been characterized as a “priority map” to help guide the allocation of both covert and overt (eye movements) attention [64]. Another model proposes that the superior and inferior parts of the PPL belong to the distinct “dorso-dorsal” stream and “ventro-dorsal” stream, respectively, with the SPL taking part in online control of action and the IPL involved in space perception and action understanding [65]. Although the current study was not designed to compare different hypotheses regarding parietal contributions to “top-down” and “bottom-up” attention, the results are consistent with the above two models that PPL deployment tends to be more inferior for exogenous orienting attention.

It has been proposed that a dynamic balance exists between the two hemispheres in orienting attention [66], [67]. Combining transcranial magnetic stimulation (TMS) and DTI, Koch and colleagues [34] found that the right PPL (intraparietal sulcus within the IPL) inhibited the activation state of the contralateral parietofrontal connection, and this effect was mediated by a transcallosal pathway located in the posterior portion of the corpus callosum. Besides, Niogi et al. [5] revealed that the orienting scores of ANT were positively linked to the mean FA values of a predefined ROI within the splenium of the corpus callosum. In line with these prior works, our findings suggest that reductions of WM connectivity between the bilateral IPL, which are primarily driven by increases in RD, reflect the reductions in the quality of orienting performance. Therefore, we expect that the important roles of the right IPL in modulating orienting attention partially depend on the myelination levels of the inter-hemispheric pathway, which might affect the unidirectional inhibitory control of the right IPL over the contralateral homologous area [34].

It is not surprising that we also detected an association between cortical thickness of the right AG and orienting performance. Using TMS on the bilateral AG and SMG, one study had found that only the right AG mediated spatial orienting during two discrete time periods after target onset [30]. By inducing activity in unilateral AG using TMS, one recent study also found increased excitability in the visual cortex, indicating that AG engages in orienting attention by its excitatory connections to the visual cortex [67]. Furthermore, lesions of the right AG are most strongly associated with spatial neglect [21], [22], in which patients fail to orient attention to the contralesional side. Significantly, the negative correlation between them suggests that thicker cortex of the right AG was associated with poor orienting attention. This is in line with previous brain structure-attention studies in normal subjects [68], [69] and patients with schizophrenia [70].

For executive control, we also found significant correlations between this component and MD/RD of the WM underlying the right SMG after correcting for multiple comparisons. Executive control is construed as the monitoring and resolution of conflict in decision making, error detection or regulation of thoughts and feelings [1]. The fronto-parietal network has been shown to be implicated in this attention system. Prefrontal regions receive information from the ventral visual pathway and play a crucial role in the conflict detection [39], [41], [71]. The PPL, as part of the dorsal visual stream, is thought to be implicated in transforming sensory information into motor outputs [37]. Recent evidence points to the recruitment of the right SMG in detecting intersensory conflict [36] and making decision [72]. PDT on the macaque parietal regions indicated that the ventral premotor cortex has a high probability of connection with the SMG [73]. It is therefore plausible that the SMG may be responsible for the conflict monitoring and vision-for action.

Notably, the TBSS statistical analysis was applied to the whole brain skeleton to localize any area of significant association with each of the three attention components without any priori hypothesis. The analysis revealed that alerting performance was significantly correlated with the FA of local WM connecting the right thalamus and supplementary motor area through the posterior limb of internal capsule. The alerting component during ANT task, which is also defined as phasic or exogenous alertness [74], represents the ability to increase response readiness subsequent to an external warning stimulus. Niogi and colleagues [5] have found that alerting performance was associated with the mean FA value in the posterior limb of internal capsule, although on the left hemisphere. Previous functional studies have identified that thalamus and supplementary motor area were both activated during the phasic alertness, irrespective of the stimulating tasks or modality [27], [75]. Besides, lesion studies also showed that patients with alerting-related impairments exhibited vascular lesions to the basal ganglia, posterior limb of internal capsule and thalamus [76] or brain functional changes involved the right supplementary motor area [77].

The intrathalamic nuclei are known to be engaged in maintaining a state of high vigilance or intrinsic alertness (a general cognitive control of arousal) [78], and the supplementary motor area is involved in movement selection and preparation [79], [80]. On one hand, the thalamic reticular nucleus receives both input from the lateral geniculate nucleus and feedback projections from the visual cortex [81]. On the other hand the supplementary motor area receives input from the ventrolateral thalamus [82]. Thus, the alerting effect may be partly mediated by the potentiation of the supplementary motor area through the thalamic “gateway” of cued attention [83].

In addition to examining the brain structural substrates for attention function, we investigated the associations between cortical morphological properties and WM parameters. Compared to the surface area, cortical thickness was more strongly related to the WM parameters, especially the FA and RD. In line with one existing study [59], we found that cortical thickness correlated negatively with FA and positively with RD in many of the parietal regions. Cortical thinning during development [84] might result from the selective elimination of synapses [85] that could refine neural circuits supporting cognitive abilities, or the proliferation of myelin into the peripheral cortical neuropil [86], and increased RD value is generally associated with WM demyelination [87]. Based on our findings, we expect that cortical thinning and FA increase is primarily driven by decrease in RD, which could further be attributed to myelination. However, such relationship was not detected in the right IPL, indicating that the positive correlations between orienting performance and both cortical thickness and FA in the right IPL are not dependent on each other.

Besides, we also revealed that cortical thickness correlated negatively with surface area within some PPL subregions, indicating that individuals with thinner cortex tend to have larger surface area [88]. Nevertheless, our findings that cortical thickness of PPL subregions, rather than surface area, is associated with attention performance, suggest that the two measures of cortical macrostructure may provide distinct information, as they are driven by distinct cellular mechanisms [89] and non-overlapping genetic factors [90]. Questions further remain concerning the cellular alterations underlying the changes of these measures and the physiological mechanisms of the various structural properties in attention function.

There are some limitations of the applied techniques that need to be addressed. First, although the AAL template had been used to define the network nodes in the previous cortical [91] and DTI [57] studies, the segmentations of the PPL may be imprecise at the boundaries of subregions due to image registration and inter-subject anatomical variability. However, because the WM skeleton is not located exclusively at lobe boundaries, these errors would have little effect on our main findings. Second, we concentrated on the relationships between attention function and the PPL structures, but we cannot exclude the possibilities of other brain regions with significant correlations. Future studies with larger population sample should be applied to further investigate the integrated influence of the grey matter and WM on the cognitive function across the whole brain.

### Conclusion

The current study evaluated MRI and DTI parameters in PPL respectively, and determined their relationships with the alerting, orienting and executive control performance of ANT in healthy individuals. The most significant novelty is the validation of the distinct roles of PPL subregions on the three sub-networks of attention. We found that orienting function was associated with the cortical thickness of the IPL and AG, and the FA underlying the right IPL. Further probabilistic tractography demonstrated that the interhemispheric WM integrity between the bilateral IPLs mediated the orienting performance. In addition, we revealed that the executive control scores were significantly associated with MD/RD of WM underlying the right SMG. Another notable finding is that alerting performance was linked to FA of local WM connecting the right thalamus and supplementary motor area through the posterior limb of internal capsule. In accordance with previous functional imaging and lesion studies, our findings will provide a better understanding of the brain structure-cognition relationships and help shed light on the current taxonomy of attention.
